# Overexpression of *OsCSP41b* Enhances Rice Tolerance to Sheath Blight Caused by *Rhizoctonia solani*

**DOI:** 10.3390/jof11080548

**Published:** 2025-07-23

**Authors:** Jianhua Zhao, Yan Zhang, Taixuan Liu, Guangda Wang, Ran Ju, Quanyi Sun, Qi Chen, Yixuan Xiong, Penfei Zhai, Wenya Xie, Zhiming Feng, Zongxiang Chen, Kemin Hu, Shimin Zuo

**Affiliations:** 1 Jiangsu Key Laboratory of Crop Genomics and Molecular Breeding/Zhongshan Biological Breeding Laboratory/Key Laboratory of Plant Functional Genomics of the Ministry of Education, Agricultural College of Yangzhou University, Yangzhou 225009, China; dx120180075@stu.yzu.edu.cn (J.Z.); mz120231357@stu.yzu.edu.cn (Y.Z.); 13554356744@163.com (T.L.); dx120210125@stu.yzu.edu.cn (G.W.); yzujuran@163.com (R.J.); sunquanyi2023@163.com (Q.S.); chenqi1099156741@outlook.com (Q.C.); opoetxiong@gmail.com (Y.X.); mx120240819@stu.yzu.edu.cn (P.Z.); wyxie@yzu.edu.cn (W.X.); fengzm@yzu.edu.cn (Z.F.); czx@yzu.edu.cn (Z.C.); hukm@yzu.edu.cn (K.H.); 2 Jiangsu Co-Innovation Center for Modern Production Technology of Grain Crops/Jiangsu Key Laboratory of Crop Genetics and Physiology, Yangzhou University, Yangzhou 225009, China; 3 Joint International Research Laboratory of Agriculture and Agri-Product Safety, Ministry of Education of China/Institutes of Agricultural Science and Technology Development, Yangzhou University, Yangzhou 225009, China

**Keywords:** rice (*Oryza sativa* L.), sheath blight, *OsCSP41b*, chloroplast

## Abstract

Sheath blight (ShB), caused by the necrotrophic fungus *Rhizoctonia solani* (*R. solani*), poses severe threats to global rice production. Developing a resistant variety with an ShB-resistance gene is one of most efficient and economical approaches to control the disease. Here, we identified a highly conserved chloroplast-localized stem-loop-binding protein encoding gene (*OsCSP41b*), which shows great potential in developing an ShB-resistant variety. *OsCSP41b*-knockout mutants exhibit chlorotic leaves and increased ShB susceptibility, whereas *OsCSP41b*-overexpressing lines (*CSP41b*-OE) display significantly enhanced resistance to *R. solani*, as well as to drought, and salinity stresses. Notably, *CSP41b*-OE lines present a completely comparable grain yield to the wild type (WT). Transcriptomic analyses reveal that chloroplast transcripts and photosynthesis-associated genes maintain observably elevated stability in *CSP41b*-OE plants versus WT plants following *R. solani* infection, which probably accounts for the enhanced ShB resistance of *CSP41b*-OE. Our findings nominate the *OsCSP41b* gene as a promising molecular target for developing a rice variety with stronger resistance to both *R. solani* and multi-abiotic stresses.

## 1. Introduction

Rice sheath blight (ShB), caused by the pathogen *Rhizoctonia solani* AG1-IA, stands as one of the most devastating rice diseases globally [1,2]. Under favorable conditions, the pathogen invades sheaths and leaves, forming characteristic lesions that impair the photosynthetic system, disrupt grain filling, and ultimately lead to severe yield reductions and quality deterioration [1,3]. Over recent decades, the widespread adoption of semi-dwarf, high-nitrogen-responsive, high-yielding rice varieties and the prevalence of intensive farming practices have aggravated the epidemic trend of ShB, resulting in annual production losses of nearly 1 million metric tons in major rice-growing regions in China [4].

Due to the persistent economic losses caused by ShB, control of the disease is becoming increasingly important. However, the conventional management of ShB disease faces a dual challenge: the application of chemical fungicides incurs high economic and environmental costs, while disease-resistant breeding progresses slowly due to the lack of germplasm resources with complete resistance [2,5]. The identified available resistance resources all exhibit partial resistance mediated by quantitative trait loci (QTL), and over 50 rice ShB-resistant QTLs have been reported [6]. Nevertheless, the polygenic nature of resistance and the complex interactions between QTLs and genetic backgrounds severely hinder the effective pyramiding of these resistant QTLs in breeding practices [2,7].

As core biotechnologies for crop genetic improvement, transgenic strategies demonstrate unique value in controlling diseases like ShB that lack natural immune resistance sources. Helliwell et al. engineered transgenic rice with pathogen-inducible ethylene synthesis by modulating the key ethylene biosynthesis gene *OsACS2*, conferring broad-spectrum resistance against both *Magnaporthe oryzae* and *R. solani* [8]. Overexpressing the osmotin gene, *OsOSM1*, significantly enhanced rice ShB resistance; and in particular, this gene is specifically induced by *R. solani* in the resistant cultivar YSBR1 and shows preferential expression in leaf sheaths during the booting stage [9]. Polygalacturonase (PG), secreted by *R. solani* during infection, is one of important pathogenic factors. Chen et al. engineered *OsPGIP1-overexpressing* rice and confirmed it was able to significantly reduce *R. solani* infection by blocking the PG-dependent virulence pathway [5]. Most recently, a CRISPR-edited *DEP1* mutant (*dep1-cys*) was developed, and the mutant enhanced both ShB resistance and yield by synchronizing ethylene/auxin signaling, breaking the resistance–productivity trade-off [10]. In addition, editing ShB-susceptible genes has also been validated as a feasible approach to developing a resistance variety without a yield penalty [11,12].

Chloroplasts, known as photosynthetic organelles, have recently been shown to play a pivotal regulatory role in plant immunity [1,13,14]. As the core pigment of chloroplasts, chlorophyll contributes to defense against *R. solani* infection: studies reveal that pathogen invasion concurrently suppresses the expression of chlorophyll biosynthesis genes while activating the expression of chlorophyll catabolism genes, thereby inducing cell death and accelerating leaf senescence. This process probably facilitates colonization by necrotrophic pathogens such as *R. solani* [1,15]. Foliar application of cytokinin effectively inhibits cell death and significantly enhances ShB resistance [14,16,17]. Collectively, these findings suggest that stabilizing chlorophyll degradation dynamics following pathogen infection may enhance rice resistance to ShB [13,18].

The chloroplast stem-loop-binding protein 41 kDa b gene (*OsCSP41b*) is essential for maintaining chloroplast function, and its loss-of-function mutants exhibit leaf chlorosis and growth retardation [19]. Studies indicate that CSP41b confers stress tolerance, as its overexpression enhances heat and salinity stress tolerance in transgenic plants [20]. Our preliminary findings revealed that *OsCSP41b* is significantly downregulated upon *R. solani* infection, suggesting its involvement in rice ShB susceptibility/resistance responses. To validate this hypothesis, here, we overexpressed the *OsCSP41b* gene in rice and found that the *OsCSP41b-overexpressing* (*CSP41b*-OE) lines not only significantly enhanced ShB resistance but also improved drought and salinity tolerance. Notably, these transgenic lines showed no alterations in plant architecture and yield-related traits compared to the wild type (WT), demonstrating high breeding potential. Transcriptome analysis suggests that *OsCSP41b* likely enhances ShB resistance by counteracting *R. solani*-induced impairment of chloroplast metabolic stability.

## 2. Materials and Methods

### 2.1. Plant Materials and Growth Conditions

Dongjin, XuDao3 (XD3), and Nipponbare (NIP) are *japonica* rice cultivars, all showing susceptibility to rice ShB. CH727 and MH63, *indica* rice cultivars, exhibit relatively high ShB resistance. Rice variety YSBR1, developed through pedigree breeding from *japonica*/*indica* hybrid progeny, displays strong resistance to *R. solani* [1]. To generate *CSP41b*-OE transgenic lines, we amplified the complete coding sequence (CDS) of *OsCSP41b* from Nipponbare cDNA and cloned it into the pCAMBIA1300U vector through homologous recombination-based assembly. Subsequently, CRISPR-mediated *OsCSP41b* mutants (*CSP41b*-KO) were generated by designing a single-guide RNA (sgRNA) against the first exon and inserting it into the pCAMBIA1305-Cas9 binary vector. Transgenic plants were produced via *Agrobacterium tumefaciens*-facilitated transformation following established methodology [21]. All primers employed appear in Table S1.

All rice seeds underwent germination at 30 °C for 3 days and were subsequently transplanted to either field plots at Yangzhou University (119°24′ E, 32°23′ N) or greenhouse conditions. For field cultivation, each rice variety was planted in experimental plots consisting of 3 rows, with 12 plants per row. Plant spacing and row spacing were maintained at 13 cm and 24 cm, respectively. For greenhouse cultivation, seedlings were grown in rectangular containers (measuring 49 cm length × 18 cm width × 15 cm depth), with five plants per container.

### 2.2. Inoculation and Evaluation of Rice ShB Resistance

The *Rhizoctonia solani* AG1-IA strain YN7, originally isolated from a rice field in Yangzhou, China, was used in the study. This strain has been widely used in previous studies [6]. Inoculum preparation: Wood veneers (0.8 mm thick) were cut into 1 cm × 2 mm slivers and placed in 9 cm diameter glass Petri dishes. After sterilization, potato dextrose broth (PDB) was added to submerge the wood pieces. A mycelial disc (8 mm diameter) of YZ19 was positioned at the dish center. Cultures were maintained at 28 °C in darkness for 3 days until wood slivers were completely covered with mycelia, after which they were ready for use.

Adult plant inoculation in greenhouse: Rice materials were cultivated in the greenhouse until the late tillering stage under controlled conditions of 28–33 °C day/night temperatures and 80–85% relative humidity. For inoculation, the embedded method was employed [6]: forceps were used to gently insert pre-cultured mycelium-colonized inoculum into the inner side of the third leaf sheath from the top. Lesion length along the sheath was measured at 21 days post-inoculation (dpi).

Inoculation of rice detached tillers in growth chamber [22]: Plants cultivated under field conditions until the booting stage were selected for tiller excision. Healthy tillers were detached from the plant, their leaves were trimmed, and they were vertically inserted into racks containing 10 tubes filled with floral foam prehydrated with Yoshida nutrient solution. After 24 h incubation in humidity-controlled chambers, inoculation was performed using the embedding method, which involved inserting a wood sliver between the leaf sheath and culm. Plants were subsequently maintained in a controlled environment (30 °C/75–95% RH, day; 28 °C/95% RH, night). Lesion length along the sheath was measured at 7 dpi using eight biological replicates (tillers per variety).

Detached leaf inoculation: Healthy rice flag leaves were inoculated following the detached leaf assay methodology of Xie et al. [14]. Briefly, flag leaves collected from field-grown plants at 25 days post-heading were surface-sterilized, trimmed into 6 cm segments, and placed on sterile moistened filter paper in 9 cm Petri dishes. Wood slivers were then applied to the center of each segment. After 7 days of incubation, disease severity was assessed by calculating the relative lesion area (RLA). This was quantified as the lesion-to-leaf area ratio using digital image analysis (Adobe Photoshop CS6, v13.0.1.3).

### 2.3. Stress and Hormone Treatments

Two-week-old rice seedlings, pre-cultured hydroponically in Yoshida solution (pH 5.8) under a 14 h photoperiod at 28 °C, were concurrently exposed to abiotic stress treatments—comprising heat stress (45 °C), cold stress (6 °C), osmotic stress (15% *w*/*v* PEG-6000), and salinity stress (150 mM NaCl) [23,24]—and foliar applications of phytohormones (50 μM kinetin, 500 μM ethephon, 50 μM indole-3-acetic acid [IAA], 50 μM gibberellic acid [GA_3_], 500 μM abscisic acid [ABA], or 0.1% Tween-20 as a surfactant control) [25]., with leaf samples collected at 5 h post-hormone treatment and 12 h post-stress exposure for immediate flash-freezing in liquid nitrogen and subsequent storage at –80 °C prior to RNA extraction.

Thirty-day-old rice seedlings were exposed to 10-day hydroponic stress treatments under controlled conditions (14 h photoperiod at 28 °C light/10 h dark at 25 °C; 65% RH), with drought simulation via Yoshida solution containing 15% (*w*/*v*) polyethylene glycol-6000 (PEG-6000), salinity stress by 150 mM NaCl amendment, and untreated controls maintained in standard Yoshida solution (pH 5.8); following treatment, surface moisture was removed prior to whole-plant fresh weight measurement using analytical balances, with statistical comparisons performed via *t*-tests (significance threshold α = 0.01) in R version 4.4.3.

### 2.4. RNA Extraction and RT-qPCR Analysis

Total RNA was isolated from leaf sheaths with TRIzol reagent (TransGen Biotech, Beijing, China). cDNA synthesis via reverse transcription employed manufacturer protocols (Vazyme, Nanjing, China). Quantitative RT-PCR analyses were conducted on a LightCycler 96 system (Roche, Basel, Switzerland) using ChamQ SYBR qPCR Master Mix (Vazyme, Nanjing, China) per manufacturer guidelines, with *UBQ5* (Os01g0328400) serving as the endogenous control. Relative gene expression levels were calculated using the 2^−ΔCt^ method. Details for all qPCR primers are in Table S1.

### 2.5. Phylogenetic Analysis

Protein sequences of the *CSP41b* gene were retrieved from Phytozome and NCBI (https://phytozome-next.jgi.doe.gov/, https://www.ncbi.nlm.nih.gov/, accessed 12 March 2025). Protein sequence alignment and trimming were performed using MEGA11 software. Phylogenetic trees were constructed via the neighbor-joining method in MEGA11 with bootstrap analysis (1000 replicates) and default parameters. Motif profiling and domain architecture of the gene family were analyzed using MEME Suite (https://meme-suite.org/meme/, accessed 15 March 2025) and NCBI CD-Search (https://www.ncbi.nlm.nih.gov/Structure/bwrpsb/bwrpsb.cgi, accessed 15 March 2025).

### 2.6. Subcellular Localization of OsCSP41b

The coding sequence of *OsCSP41b* was PCR-amplified and subcloned into the PAN580-GFP expression vector. For each transformation, 5–10 μg of both PAN580-GFP and recombinant OsCSP41b-GFP plasmids were aliquoted into 2 mL centrifuge tubes. Subsequently, 100 μL of pre-prepared protoplast solution was added with vigorous vortexing, followed by addition of 110 μL PEG (polyethylene glycol) solution and gentle tube tapping to ensure homogenization. The mixture underwent 10-min dark incubation at room temperature. After adding 440 μL W5 solution with brief mixing, samples were centrifuged (200× *g*, 3 min) and supernatants aspirated. Pelleted protoplasts were resuspended in 300 μL WI solution (composition: 0.5 M mannitol, 20 mM KCl, 4 mM MES; pH 5.7) and maintained under light/dark conditions at 25 °C for 6–16 h. Subcellular localization was examined using an LSM880 confocal laser scanning microscope (Zeiss, Shanghai, China) at Yangzhou University’s Core Facilities.

### 2.7. Protein Extraction and Western Blot Analysis

Total protein was extracted from leaves of three-week-old T1 transgenic and WT rice plants. Leaf tissues were pulverized in liquid nitrogen. The powder was homogenized in ice-cold extraction buffer (50 mmol/L Tris–HCl, 150 mmol/L NaCl, 0.5% [*w*/*v*] SDS, pH 7.5) for 3 h at 4 °C with constant stirring. The crude lysate was centrifuged at 12,000 rpm for 10 min at 4 °C, and the supernatant was collected as the total protein fraction.

Protein samples were separated by 12% sodium dodecyl sulphate–polyacrylamide gel electrophoresis (SDS-PAGE) and then transferred to a nitrocellulose membrane. The membrane was blocked with 5% skimmed milk in phosphate-buffered saline with Tween-20 (PBST) buffer for 2 h and washed 3 times with PBST. It was then incubated with anti-Flag antibody (AE004, ABclonal, Wuhan, China) at a 1:5000 dilution. An anti-Actin antibody (1:5000, AC009, ABclonal, Wuhan, China) was used to detect rice Actin as an internal control. Finally, West Pico PLUS chemiluminescent substrate (Solabao, Beijing, China) was added for a 10-min reaction, and images were captured using an Amersham Imager 600 imager (General Electric, Cincinnati, OH, USA).

### 2.8. Chlorophyll Content Measurement

Chlorophyll extraction was performed on greenhouse-grown 3-week-old rice plants. Leaf tissues were triturated under dim green light in ice-cold 80% acetone (*v*/*v*), followed by centrifugation at 4 °C to pellet plant debris. Spectrophotometric analysis of supernatants was conducted at visible wavelengths (400–700 nm). Chlorophyll quantification employed the formulas established by Lichtenthaler [26], with total chlorophyll concentrations normalized against fresh leaf mass.

### 2.9. Evaluation of Agronomic Traits in the Field

Comprehensive agronomic trait evaluation was conducted under standardized field conditions at Yangzhou University. *CSP41b*-OE and WT plants were cultivated in six-row plots with 11 plants per row (13 cm × 24 cm spacing) using three biological replicates. At maturing stage, 18 plants per genotype from two central rows were evaluated for the following: plant height, flag leaf length, panicle number per plant (PNP), grain length and width, 1000-grain weight (TGW), seed number per panicle (SNP), seed-setting rate = (filled grains per main panicle/total grains per main panicle) × 100% (SSR), and yield per plant = PNP × SNP × SSR × TGW.

### 2.10. RNA-Seq and Data Analysis

For RNA-seq analysis, *CSP41b*-OE and WT plants were greenhouse-cultivated in containers (49 × 18 × 15 cm; 5 plants/container) under controlled conditions (28 °C/80% RH, 14-h light). At late tillering stage, plants were inoculated according to the adult plant inoculation in greenhouse protocol. At 24 h post-inoculation (hpi), inoculated and control leaf sheath samples from both *CSP41b*-OE and WT plants were collected with three biological replicates per sample and immediately flash-frozen in liquid nitrogen, followed by total RNA extraction using TRIzol reagent (TransGen Biotech, Beijing, China); subsequent RNA sequencing was performed on the BGI T7 platform at Benagen Tech Inc. (Wuhan, China), and data analysis involved mapping of processed reads to the IRGSP-1.0 reference genome using HISAT2 v2.2.1, gene quantification via StringTie v2.2.3, and screening of differentially expressed genes (DEGs) with DESeq2 v3.21 (R v4.4.3) using thresholds of FDR-adjusted *p* < 0.01 and |log_2_(fold change)| ≥ 1.

## 3. Results

### 3.1. CSP41b Is Highly Conserved Among Plants and Suppressed Expression by R. solani Infection

**Figure 1:**
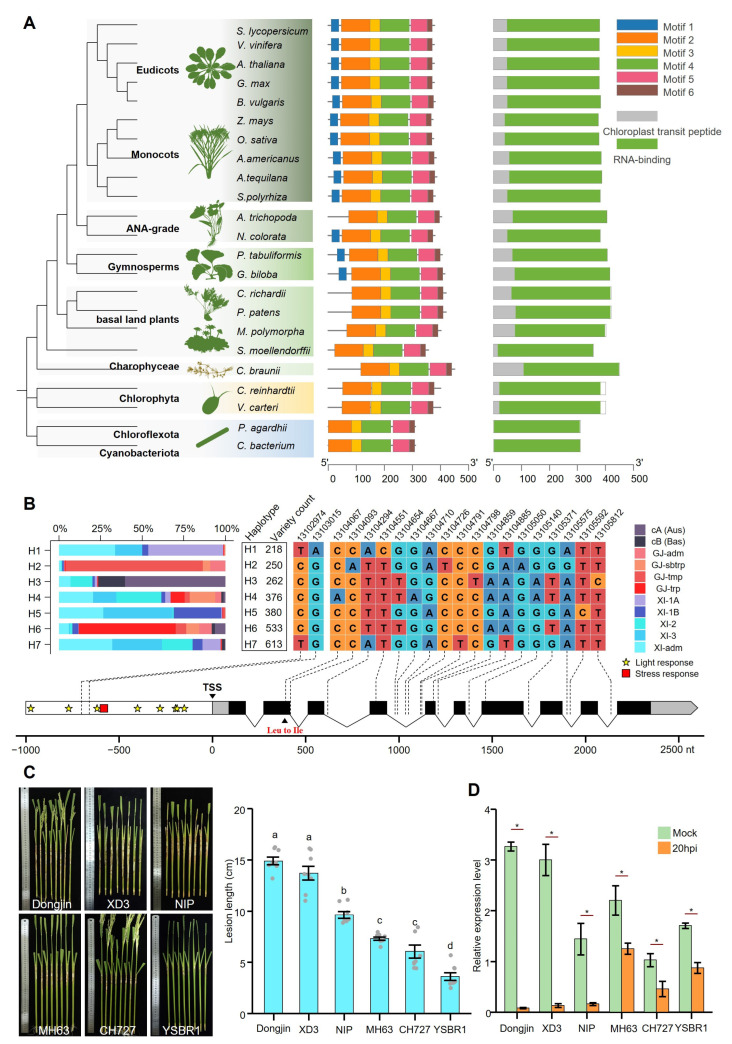
Phylogenetic analysis of CSP41b protein among plants and *OsCSP41b* expression change due to *R. solani* infection. (**A**) Phylogenetic tree, motif analysis, and domain architecture of the CSP41b protein in photosynthetic organisms. (**B**) Haplotype analysis of *OsCSP41b* in the 3K Rice Genome Project. The upper panel shows the percentage distribution of rice subpopulations for different haplotypes and their corresponding SNP combinations. The bottom panel shows the locations of individual SNPs within the *OsCSP41b* gene and its 1000 bp upstream region, where white rectangles represent promoter, gray rectangles represent UTR, and black rectangles represent CDS. (**C**) Comparison of lesion lengths on detached tillers from different rice cultivars inoculated with *R. solani* (7 dpi). Values are presented as mean ± standard error (SE) (*n* = 8). Different letters indicate statistically significant differences as determined by the one-way analysis of variance (ANOVA) followed by least significant difference (LSD) test (*p* < 0.05). (**D**) Effect of *R. solani* inoculation on *OsCSP41b* expression levels at 20 hpi in greenhouse-cultivated plants. Values represent mean ± SE (*n* = 3). Significant differences determined by two-tailed Student’s *t*-test (* *p* < 0.05).

BLASTP homology searches revealed that the CSP41b protein is fundamentally conserved as a near-ubiquitous single-copy protein across photosynthetic lineages, spanning cyanobacteria, algae, basal land plants, and seed plants. The phylogenetic analysis reveals that CSP41b originated in photosynthetic bacteria, transferred to early algae via endosymbiotic events, and ultimately became widely conserved in plant genomes (Figure 1A). The protein sequence alignment demonstrates minimal divergence, except for the acquisition of chloroplast transit peptides in eukaryotes (Figure S1). The core RNA-binding domain exhibits remarkable conservation (average sequence similarity = 85%) across cyanobacteria, green algae, and higher plants, implying an evolutionarily conserved role of CSP41b in regulating RNA metabolism within photosynthetic organelles. These findings suggest that this protein serves as an irreplaceable regulator sustaining homeostasis in chloroplast gene expression.

Furthermore, we analyzed variations of the *OsCSP41b* gene within the 3K Rice Genomes [27]. The haplotype analysis identified seven major haplotypes spanning the gene and its 1 kb upstream promoter (Figure 1B). Only haplotype H4 exhibits a C-to-A transversion at chromosome 12 position 13,104,067, causing a leucine-to-isoleucine substitution. No amino acid changes occurred in the other six haplotypes. The promoter analysis revealed eight light-responsive cis-elements and one stress-responsive cis-element. Remarkably, no SNPs were present within these cis-elements. These findings collectively suggest that *OsCSP41b* is functionally highly conserved across the rice population and is essential for rice survival.

Previously, we noticed that the expression of *OsCSP41b* is suppressed during *R. solani* infection [1]. To validate this expression response across differential resistance backgrounds, six rice varieties were selected: three susceptible types (Dongjin, XD3, Nipponbare) and three resistant types (MH63, CH727, YSBR1). In a detached tillers inoculation assay, we confirmed that Dongjin was the most susceptible (lesion length: 14.9 ± 0.4 cm), followed by XD3 (13.7 ± 0.7 cm) and Nipponbare (9.6 ± 0.3 cm). YSBR1 exhibited the strongest resistance (3.6 ± 0.4 cm), followed by CH727 (6.0 ± 0.5 cm) and MH63 (7.3 ± 0.2 cm) (Figure 1C). Reverse transcription quantitative real-time PCR (RT-qPCR) revealed *OsCSP41b* expression patterns across these varieties. At 20 hpi, *OsCSP41b* expression was significantly suppressed in all the varieties. Notably, relatively susceptible varieties exhibited stronger suppression, with expression reduced to as little as 2.5% of the mock controls, while relatively resistant varieties showed weaker suppression at approximately 50% of the control levels (Figure 1D), suggesting a positive regulation of *OsCSP41b* in ShB resistance.

### 3.2. Expression Pattern and Subcellular Localization of OsCSP41b in Rice

**Figure 2:**
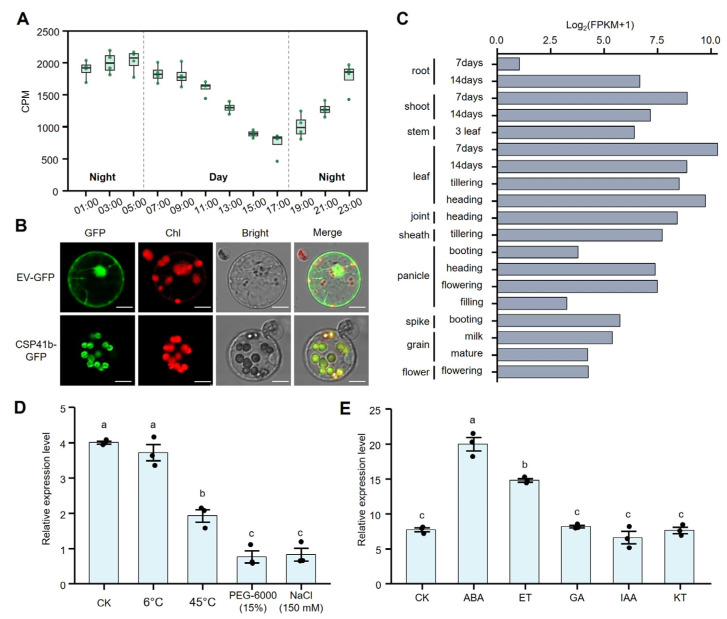
Expression patterns and subcellular localization of OsCSP41b. (**A**) Diurnal expression changes of *OsCSP41b* over a 24 h period (*n* = 4). Data are presented as box plots. CPM: Counts Per Million. (**B**) Subcellular localization of OsCSP41b in rice protoplasts. The CSP41b-GFP fusion protein localizes to chloroplasts. Bar = 10 μm. EV: Empty Vector, GFP: Green Fluorescent Protein. (**C**) Expression levels of *OsCSP41b* in various tissues at distinct developmental stages. FPKM: Fragments Per Kilobase Million. (**D**) Relative expression levels of *OsCSP41b* under different abiotic stress treatments. (**E**) Relative expression levels of *OsCSP41b* following phytohormone treatments. Values are presented as mean ± SE (*n* = 3). Different letters indicate statistically significant differences, as determined by the one-way ANOVA followed by LSD test (*p* < 0.05).

Chloroplast-related genes generally demonstrate responsiveness to photoperiodic cycles [28]. Through searching publicly available transcriptome data [29], we further analyzed the diurnal expression rhythm of *OsCSP41b*. We found that the gene exhibited dark-induced upregulation, with expression persistently rising upon dark shift and gradually declining upon light restoration. The peak expression at night was approximately twofold higher than the minimum level in daytime (Figure 2A). The predicted N-terminal chloroplast transit peptide suggested the potential chloroplast localization of OsCSP41b (Figure 1D). Furthermore, we constructed PAN580-CSP41b-GFP and control PAN580-GFP vectors for the transient transformation of rice protoplasts, and found that the CSP41b-GFP signal showed unequivocal co-localization with chloroplast fluorescence (Figure 2B). Together, these data confirm the chloroplast-targeting property of OsCSP41b.

Using the spatiotemporal expression data from MBKbase [30], we found that *OsCSP41b* expression is constitutive across all rice organs and developmental stages, while demonstrating predominant expression in leaves higher than in other tissues (Figure 2C). To functionally characterize this expression pattern, we quantified *OsCSP41b* responses to abiotic stresses and phytohormones in Nipponbare seedlings at 12 h post-treatment via qPCR. We found that cold stress exerted no significant effect on *OsCSP41b* expression, whereas heat, drought, and salinity stresses triggered pronounced downregulation of *OsCSP41b* expression (Figure 2D). At 5 h post-hormone treatment, *OsCSP41b* expression was significantly upregulated in response to abscisic acid (ABA) and ethylene (ET). By contrast, the gibberellin (GA), auxin (IAA), and cytokinin (KT) treatments elicited no substantial alterations (Figure 2E).

### 3.3. Knockout of OsCSP41b Significantly Affects Rice Growth While Its Overexpression Displays Comparable Phenotype and Yield-Associated Traits to the Wild Type

**Figure 3:**
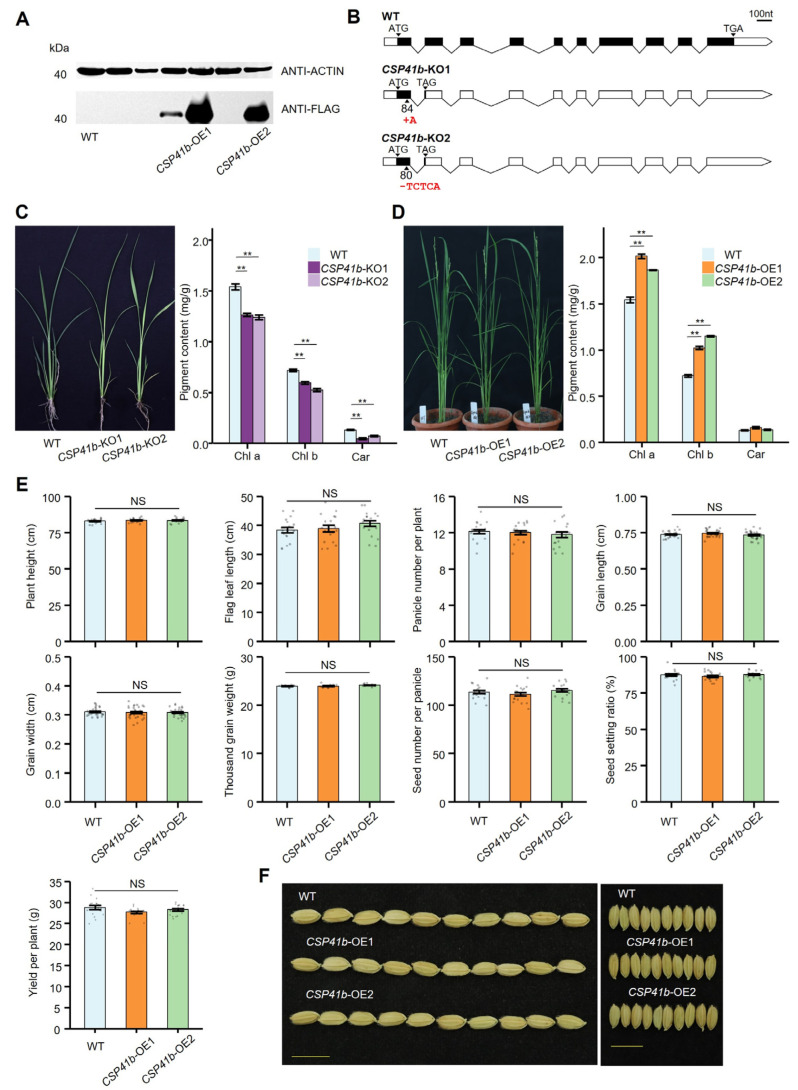
Generation of *OsCSP41b* overexpression and knockout lines. (**A**) Detection of CSP41b-Flag fusion protein in WT and *CSP41b*-OE plants by Western blotting. Out of six overexpression lines, high expression of *OsCSP41b* was detected in two lines, which were named *CSP41b*-OE1 and *CSP41b*-OE2. (**B**) Genotyping confirmation of targeted mutations in two Os*CSP41b* knockout lines. Schematic illustrates the CRISPR target sites. (**C**) Chlorophyll content in leaves of two independent *CSP41b*-OE lines (*n* = 3). Chla, Chlb, and Car represent chlorophyll a, chlorophyll b, and carotene. (**D**) Leaf chlorophyll content in *CSP41b*-KO lines versus WT controls (*n* = 3). (**E**) Bar plot shows plant height (*n* = 18), flag leaf length (*n* = 18), panicle number per plant (*n* = 18), grain length (*n* = 30), grain width (*n* = 30), thousand-grain weight (*n* = 10), seed number per panicle (*n* = 18), seed setting ratio (*n* = 18), and yield per plant (*n* = 18) in WT versus *CSP41b*-OE plants. (**F**) Grain phenotype comparison between WT and *CSP41b*-OE plants. Bar = 1 cm. Values are presented as mean ± SE. Significant differences were determined using a two-tailed Student’s *t*-test (** *p* < 0.01). NS: No Significance.

To determine the function of *OsCSP41b* in rice development, we generated two independent *OsCSP41b*-overexpressing lines (*CSP41b*-OE1, *CSP41b*-OE2), and two independent knockout lines (*CSP41b*-KO1, *CSP41b*-KO2), in the Nipponbare background. A Western blot analysis confirmed a significant increase in OsCSP41b protein levels in two *CSP41b*-OE lines among six independent overexpressing lines (Figure 3A and Figure S2). Through sequencing, two *CSP41b*-KO mutants were confirmed as carrying 1 bp and 5 bp deletions, respectively (Figure S3). Premature termination codons were identified in the *OsCSP41b* sequences of both mutants, resulting in truncated RNA-binding domains (Figure 3B).

Consistent with the *OsCSP41b* mutants identified by a previous study [19], both *CSP41b*-KO lines displayed yellow-leaf phenotypes accompanied by significantly reduced chlorophyll content relative to the WT (Figure 3C). However, almost no appearance difference was observed between the *CSP41b*-OE lines and the WT plants in the field. Since a clearly reduced chlorophyll content was observed in the *CSP41b*-KO lines, we then measured the chlorophyll contents for the *CSP41b*-OE lines. We found that compared to the WT plants, both *CSP41b*-OE lines displayed a significant increase in chlorophyll a and chlorophyll b, but no significant difference in carotene content (Figure 3D). In addition, a total of eight agronomic and yield-related traits were measured, and all showed no significant differences from the WT. As expected, the *CSP41b*-OE lines showed comparable plant yield (PY), calculated by panicle number per plant (PNP), seed number per panicle (SNP), seed setting ratio (SSR), and thousand grain weight (TGW) to the WT (Figure 3E). Altogether, we conclude that OsCSP41b is required for normal rice development, and its overexpression does not affect rice growth and grain yield.

### 3.4. OsCSP41b Overexpression Enhances Rice Tolerance to R. solani, as Well as to Drought and Salinity

**Figure 4:**
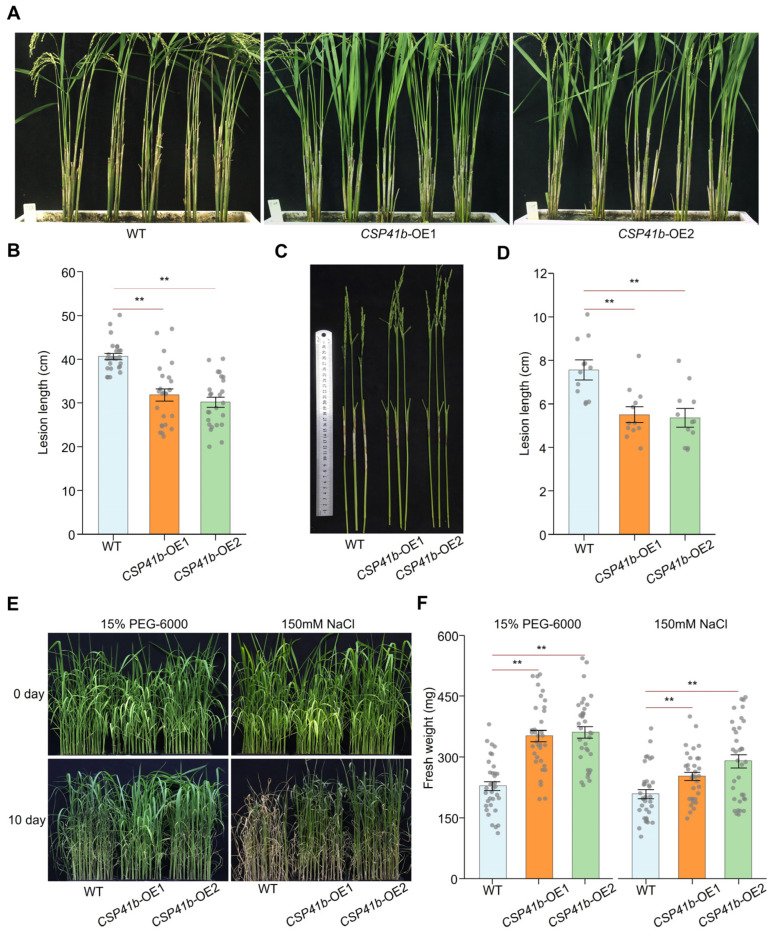
Overexpression of *OsCSP41b* enhances rice resistance to *R. solani* and tolerance to abiotic stresses. (**A**,**B**) Disease evaluation of rice plants in adult plant inoculation assay in greenhouse. Disease symptoms were assessed at 21 dpi with *R. solani* (*n* = 25). (**C**,**D**) Disease evaluation of rice plants in detached tiller inoculation assay in growth chamber. Lesion length was measured at 7 dpi (*n* = 12). (**E**,**F**) Phenotypic responses of *CSP41b*-OE and WT plants to drought and salinity stresses (*n* = 35). Fresh weights of different rice lines were compared 10 days post-treatment. Values are presented as mean ± SE. Significant differences were determined using a two-tailed Student’s *t*-test (** *p* < 0.01).

To further determine the role of *OsCSP41b* in rice tolerance to *R. solani* and abiotic stresses, we firstly inoculated the *OsCSP41b*-related transgenic rice lines with *R. solani*. As shown in Figure 4A,B, we found that lesion lengths of two *CSP41b*-OE lines reached 31.8 ± 1.4 cm and 30.2 ± 1.1 cm at 21 dpi, showing significant reduction compared to WT plants (40.6 ± 0.7 cm) in the greenhouse adult-plant inoculation assay. Similarly, in the detached tillers inoculation assay in the growth chamber, the lesions in the *CSP41b*-OE lines reached 5.5 ± 0.4 cm and 5.4 ± 0.4 cm at 7 dpi, significantly shorter than the WT (7.6 ± 0.5 cm) (Figure 4C,D). By contrast, the two independent *CSP41b*-KO lines exhibited severely com-promised resistance to *R. solani* compared to the WT plants in both detached leaf and tiller inoculation assays (Figure S4). These data indicate that the overexpression of *OsCSP41b* is able to increase rice ShB resistance.

Since *OsCSP41b* transcription is also affected by drought and salt stress, we further evaluated stress tolerance in *CSP41b*-OE lines. Seedlings at 30 days post-germination were exposed to drought stress (15% *w*/*v* PEG-6000) and salinity stress (150 mM NaCl) for 10 days. Following the treatment, both *CSP41b-OE* lines demonstrated significantly enhanced stress tolerance (Figure 4E). The WT plants exhibited average fresh weights of 229 ± 11 mg (drought) and 209 ± 11 mg (salinity), whereas the *CSP41b*-OE lines recorded 353 ± 14 mg and 362 ± 14 mg under drought stress, and 253 ± 10 mg and 290 ± 16 mg under salinity stress, respectively (Figure 4F). Conversely, the knockout of *OsCSP41b* significantly compromised tolerance to both drought and salt stresses.

Overall, we conclude that *OsCSP41b* overexpression confers dual-enhanced stress tolerance in rice, significantly suppressing ShB progression and increasing biomass accumulation under drought/salinity stress compared to the WT.

### 3.5. Chloroplast Transcripts and Photosynthesis-Associated Genes Show More Stabilization in CSP41b-OE Lines than WT After Infection with R. solani

**Figure 5:**
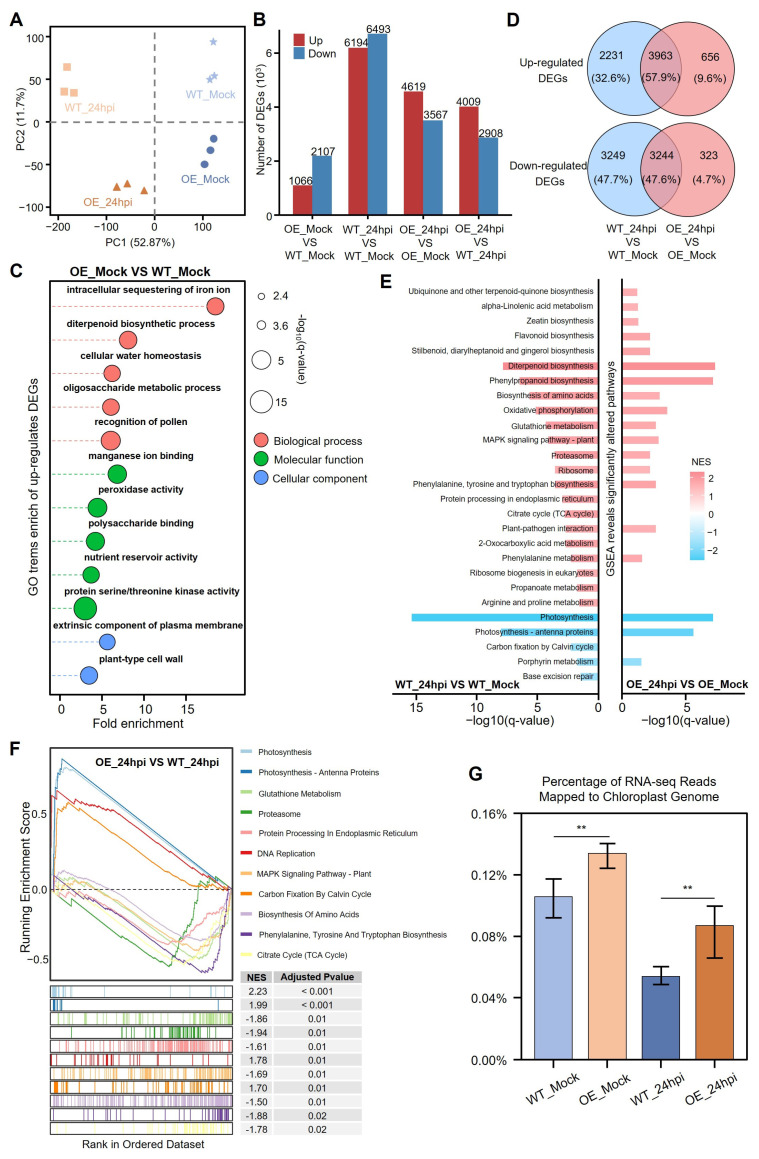
Transcriptomic dynamics in *CSP41b*-OE versus WT plants pre- and post-inoculation. (**A**) Principal component analysis (PCA) of gene expression in 12 samples across four experimental groups. Samples within the same group are denoted by same symbols. (**B**) Bar plot quantifying the number of DEGs between indicated group comparisons. (**C**) GO enrichment of upregulated DEGs in OE lines between WT plants. (**D**) The Venn diagram illustrates overlaps of DEGs between OE lines and WT plants before and after *R. solani* infection. (**E**) Gene Set Enrichment Analysis (GSEA) of DEGs from WT_24hpi vs. WT_Mock and OE_24hpi vs. OE_Mock comparisons. Enriched KEGG pathways shown on y-axis, significance levels on x-axis. Enrichment scores are color-coded (red: upregulated; blue: downregulated; NES: Normalized Enrichment Score). (**F**) GSEA for DEGs from OE_24hpi vs. WT_24hpi comparison: Top left: Enrichment score (ES) profile; Bottom left: Barcode plot showing positions of pathway-specific genes in ranked DEG list (colors represent distinct gene sets). (**G**) Proportion of reads from chloroplast genome relative to total reads across groups. Values presented as bar plot (mean ± SE). Significant differences were determined using a two-tailed Student’s *t*-test (** *p* < 0.01).

To clarify the mechanism of *CSP41b*-OE against *R. solani* in rice, we performed a comparative transcriptomic profiling of leaf sheaths from *CSP41b*-OE1 and WT plants at 24 hpi with *R. solani*. A principal component analysis (PCA) delineated four non-overlapping clusters across the biological replicates, demonstrating consistent group segregation (Figure 5A). To confirm the reliability of the RNA-seq data, the expression of five pathogenesis-related genes selected from the analysis was independently validated using RT-qPCR (Figure S5). Following *R. solani* infection, the RNA-seq analysis identified 13,407 DEGs in the WT plants, with 6914 upregulated and 6493 downregulated (Figure 5B). In contrast, the *CSP41b*-OE plants exhibited only 8186 DEGs, with 4619 upregulated and 3567 downregulated, representing a 39% reduction compared to the WT. This attenuated transcriptional response suggests that the *CSP41b*-OE plants experienced a significantly reduced impact from the pathogen.

Among the unchallenged healthy plants, the *CSP41b*-OE plants exhibited 3173 DEGs compared to the WT, with 1066 upregulated and 2107 downregulated genes (Figure 5B). The gene ontology (GO) enrichment analysis demonstrated that the upregulated DEGs were significantly enriched in five core defense mechanisms: intracellular iron ion sequestration to deprive pathogens of essential nutrients, diterpenoid biosynthesis for producing antimicrobial compounds (e.g., momilactones) that directly suppress pathogen growth, peroxidase activity reinforcing cell wall integrity and scavenging reactive oxygen species (ROS), manganese ion binding facilitating antioxidant enzyme function and structural fortification, and serine/threonine kinase activity modulating immune signaling cascades to amplify disease resistance. These collectively enhanced antioxidant capacity and multifaceted stress responsiveness in rice against pathogens. Additionally, the GO term cellular water homeostasis was found to be significantly enriched, containing nine wall-associated receptor-like kinase genes that play a critical role in biotic and abiotic stress responses, supporting the enhanced drought and salinity tolerance observed in the *CSP41b*-OE lines (Figure 5C).

We further compared ShB-induced DEGs between the *CSP41b*-OE lines and the WT plants, revealing 7207 common DEGs with consistent expression patterns across both genotypes. Notably, the *CSP41b*-OE lines exhibited 656 specifically upregulated DEGs (29.4% of WT-specific upregulated DEGs) and 323 specifically downregulated DEGs (9.9% of WT-specific downregulated DEGs) (Figure 5D). To gain deeper insights, a gene set enrichment analysis (GSEA) was performed on the DEGs to identify significantly altered Kyoto encyclopedia of genes and genome (KEGG) pathways. The analysis revealed a total of 27 significantly impacted pathways, 13 of which were uniquely present in either the *CSP41b*-OE or the WT plants. Notably, five upregulated pathways were identified exclusively in the *CSP41b*-OE plants: (1) ubiquinone/terpenoid-quinone biosynthesis may participate in scavenging pathogen-induced ROS [31]; (2) α-linolenic acid metabolism amplified jasmonate signaling to induce antifungal defensins and hydrolase inhibitors [32]; (3) zeatin biosynthesis delayed senescence during *R. solani* infection [14]; (4) metabolites derived from the flavonoid biosynthesis, including sakuranetin, confer resistance against pathogen infection [33]; and (5) stilbenoid/diarylheptanoid biosynthesis generated phytoalexins like deoxyrhapontigenin that inhibited hyphal growth (Figure 5E) [34]. These specific DEGs demonstrate that *CSP41b*-OE lines are able to orchestrate precise immune activation while minimizing global transcriptional disruption.

Finally, we compared *CSP41b*-OE to WT plants at 24 hpi, identifying a total of 6917 DEGs. Among these, 4009 DEGs were upregulated and 2908 DEGs were downregulated (Figure 5B). The GSEA analysis identified 11 significantly impacted pathways, among which four pathways were found to be upregulated: photosynthesis, photosynthesis–antenna proteins, DNA replication, and carbon fixation by Calvin cycle (Figure 5F). We further investigated alterations in photosynthesis-associated genes. Screening the curated FunRiceGenes database of 180 cloned photosynthetic genes revealed that 83 exhibited differential expression between the *CSP41b*-OE and WT plants at 24 hpi (Figure S6) [35]. Despite *R. solani* infection causing an overall decline in the expression of photosynthesis-related genes in both *CSP41b*-OE and WT plants, 68 DEGs exhibited higher expression levels in the *CSP41b*-OE compared to the WT, with 11 showing the most substantial alterations. These DEGs functionally encompass chloroplast gene expression regulation (*ASL1*, *CDE4*, *OsWSL3*), carbon assimilation and energy metabolism (*Osppc4*, *FPPS1*), photo-oxidative defense systems (*OsHI-LOX*, *CDSP32*, *OsFLU1*), pigment metabolic stability (*WFSL1, NOL*), and photosynthetic electron transport chain components (*CRR6*). Based on these findings, we further quantified the proportion of RNA-seq reads mapped to the chloroplast genome. Our analysis revealed that although *R. solani* infection suppressed chloroplast gene expression in both *CSP41b*-OE and WT plants, transcriptional levels remained significantly elevated in the *CSP41b*-OE compared to the WT, which demonstrated enhanced basal and infection-responsive robustness at both pre- and post-infection (Figure 5G). Notably, key genes encoding ribosomal proteins (e.g., *rps3*, *rps11*, *rps12*, *rps14*, *rps19*), RNA polymerases (*rpoA*), and photosystem components (e.g., *ndhD*, *psaB*) displayed significantly elevated expression in the *CSP41b*-OE plants compared to the WT following *R. solani* infection (Figure S7).

Together, these data demonstrate that *CSP41b*-OE plants have a stronger ability to suppress the effects of the destruction of *R. solani* infection on chloroplast- and photosynthesis-associated gene expression and ultimately enhance resistance to ShB disease.

## 4. Discussion

Breeding rice varieties resistant to ShB (SB) using QTL faces significant challenges, primarily due to the complex polygenic nature of this resistance. Although extensive studies on ShB-resistant QTLs have been conducted, the number of QTLs functionally validated under field conditions remains fewer than ten. Furthermore, the epistatic interactions between these QTLs introduce additional difficulties in breeding. Enhancing rice resistance to ShB through the regulation of resistance-response genes has been widely reported. However, only a few genes, such as *OsPGIP1*, *OsACS2*, *tlp-D34*, *chill*, *RCH10*, and *DEP1*, have been demonstrated to improve resistance to ShB under field conditions [5,8,9,10,36,37]. Previous studies have indicated that chlorophyll degradation may be a critical susceptibility response in rice and maize during *R. solani* infection. Maintaining chlorophyll levels may play a general role in protecting crops against necrotrophic pathogens like *R. solani*. Our study found that the overexpression of the *OsCSP41b* gene enhances rice resistance to ShB, as well as drought and salt stresses, without adversely affecting growth or grain yield, demonstrating the breeding potential of *OsCSP41b* in engineering ShB-resistant varieties.

Increasing research emphasizes that chloroplasts not only serve as essential organelles for photosynthesis but also function as pivotal hubs in plant immunity, orchestrating immune activation and growth–defense trade-offs by modulating calcium (Ca^2+^) signaling, ROS accumulation, hormone biosynthesis, stromule-mediated chloroplast–nuclear communication, and retrograde signaling [15,38,39,40,41]. Plant pathogens may secrete effectors to target chloroplasts for reducing host defenses for infection. For example, *Pseudomonas syringae* secretes the effector HopN1 to degrade the photosystem II (PSII) subunit PsbQ, thereby compromising photosynthetic efficiency and suppressing chloroplastic ROS generation [42], while the *Magnaporthe oryzae* effector MoApx1 specifically binds the PSI core component OsPsaD, blocking electron transport and suppressing ROS generation [43]. Distinct studies by Xie et al. and Cao et al. respectively identified chlorophyll metabolism-associated genes, *OsNYC3* and *OsSGR*, involved in rice disease resistance. They found that knocking out or suppressing these genes’ expression increased chlorophyll content, delayed leaf senescence, and enhanced rice resistance against ShB. Furthermore, it was inferred that the elevated cytokinin accumulation in *OsSGR* knockout lines accounted for the enhanced ShB resistance [1,14]. Ghosh et al. demonstrated that *R. solani* infection suppresses host photosynthesis, induces ROS accumulation, and promotes cell death, thereby enhancing rice susceptibility to sheath blight [44]. Cao et al. identified five chloroplast-targeting secreted proteins from a *R. solani* yeast library via screening with ShB-resistant chloroplast proteins. These proteins induce plant cell death, indicating chloroplast-mediated manipulation of host cellular processes [45]. Collectively, these findings demonstrate that suppressing the photosynthetic system is one of important steps during *R. solani* infection in rice.

In this study, we found that *CSP41b*-OE lines under *R. solani* infection similarly elevate zeatin biosynthesis, one of the key cytokinin components, further emphasizing the critical role of chlorophyll and cytokinin in regulating rice ShB resistance. OsCSP41b is a critical factor for maintaining normal plant growth and chloroplast gene expression. The absence of this protein leads to reduced stability in key chloroplast RNAs, such as 16S rRNA, 23S rRNA, and mRNAs encoding photosynthetic proteins. Previous studies showed that the OsCSP41 complex plays a key role in stabilizing untranslated RNAs during the night in response to the chloroplast redox state, while dissociating under light to coordinate its function with ribosomes. Furthermore, OsCSP41b enhances the transcriptional activity of plastid RNA polymerase and participates in polysome formation by stabilizing ribosome assembly intermediates [46,47,48]. Here, after *R. solani* infection, we found that chloroplast RNA stability, particularly for key genes involved in photosynthesis, was effectively maintained in the *CSP41b*-OE plants, suggesting that maintaining the stability of chloroplast gene expression is critically important for rice resistance to *R. solani*. As a result, we conclude that the enhanced resistance of the *CSP41b*-OE line against ShB is mainly attributable to its stronger ability to suppress the effects of the destruction of *R. solani* infection on chloroplast- and photosynthesis-associated gene expression.

Critically, the observed transcriptomic responses occurred under controlled conditions. While providing mechanistic insights, real-world agroecosystem stresses may moderate these effects. Nevertheless, *CSP41b*-OE enhances basal defense readiness and optimizes response deployment, requiring fewer differentially expressed genes during *R. solani* infection. This confers practical advantages, including accelerated early defense, efficient anti-pathogen resource allocation, and improved growth–defense balance, ultimately promoting field-level plant health through reduced disease losses and immunity-caused growth suppression. To leverage this, broad-spectrum germplasm screening across ecotypes is essential to identify elite haplotypes for pyramiding, translating mechanistic findings into durable field resistance.

Rice, as a globally cultivated staple crop, faces severe challenges from abiotic stresses like salt and drought, which significantly impair growth and yield. Recent syntheses reveal that rice primarily relies on three core systems to combat these stresses: (1) osmotic adjustment through upregulated *OsP5CS* and *OsTPS1* expression, accumulating osmolytes (e.g., proline, trehalose) to sustain cellular water homeostasis; (2) ion homeostasis regulation mediated by high-affinity K^+^ transporters (*OsHKT1;5*, *OsHKT2;1*, *OsHKT1;1*) critical for K^+^/Na^+^ homeostasis under salinity; and (3) enhanced antioxidant activity that counteracts ROS accumulation under drought/salinity through increased expression and activity of enzymes like SOD, CAT, and APX. Additionally, transcription factors (DREBs, NACs, bZIPs) and hormones (abscisic acid, gibberellins) orchestrate these responses [49]. OsCSP41b contributes indirectly to stress resilience while functioning not directly in drought/salt tolerance but as a guardian for chloroplast RNA stability, boosting multi-stress resistance without compromising agronomic traits. Therefore, the reported stress-tolerance genes could be functionally combined with *OsCSP41b* overexpression to further enhance multi-stress resistance in rice. This unique mode positions *OsCSP41b* as a prime candidate for developing rice varieties with concurrent salt/drought tolerance.

## Data Availability

The original RNA-seq data were deposited in the Genome Sequence Archive of the China National Center for Bioinformation (CNCB) with GSA ID: CRA026490.

## Supplementary Materials

The following supporting information can be downloaded at https://www.mdpi.com/article/10.3390/jof11080548/s1. Figure S1: Protein sequences used for constructing the phylogenetic tree of the CSP41b in photosynthetic organisms; Figure S2: Expression OsCSP41b-FLAG protein in WT and *CSP41b*-OE plants; Figure S3: Sequencing chromatograms near the target sites of the two knockout lines of *OsCSP41b*; Figure S4: Relative lesion area measurement on detached leaves at 7 dpi; Figure S5: Five randomly selected pathogen-responsive rice genes were subjected to qPCR to validate the reliability of transcriptome data; Figure S6: Heatmap of nuclear genome-encoded photosynthesis-related DEGs; Figure S7: Heatmap showing DEGs mapped to the chloroplast genome across all samples; Table S1: The primer sequence information involved in this article.

## Abbreviations

The following abbreviations are used in this manuscript:

ShB	Sheath blight
OsCSP41b	Chloroplast stem-loop-binding protein of 41 kDa b
WT	Wild type
OE	Overexpressing
KO	Knockout
dpi	Days post-inoculation
hpi	Hours post-inoculation
PCA	Principal component analysis
DEGs	Differentially expressed genes
GSEA	Gene set enrichment analysis
GO	Gene ontology
KEGG	Kyoto encyclopedia of genes and genome
QTL	Quantitative trait loci
ROS	Reactive oxygen species
