# Supplementary material for: Overexpression of OsCSP41b Enhances Rice Tolerance to Sheath Blight Caused by Rhizoctonia solani
Source: J Fungi (Basel). 2025 Jul 23;11(8):548. doi: 10.3390/jof11080548 (PMC12387978; doi:10.3390/jof11080548)
Supplement: Supplementary file 1 [file jof-11-00548-s001.zip › jof-3753982-supplementary.pdf]

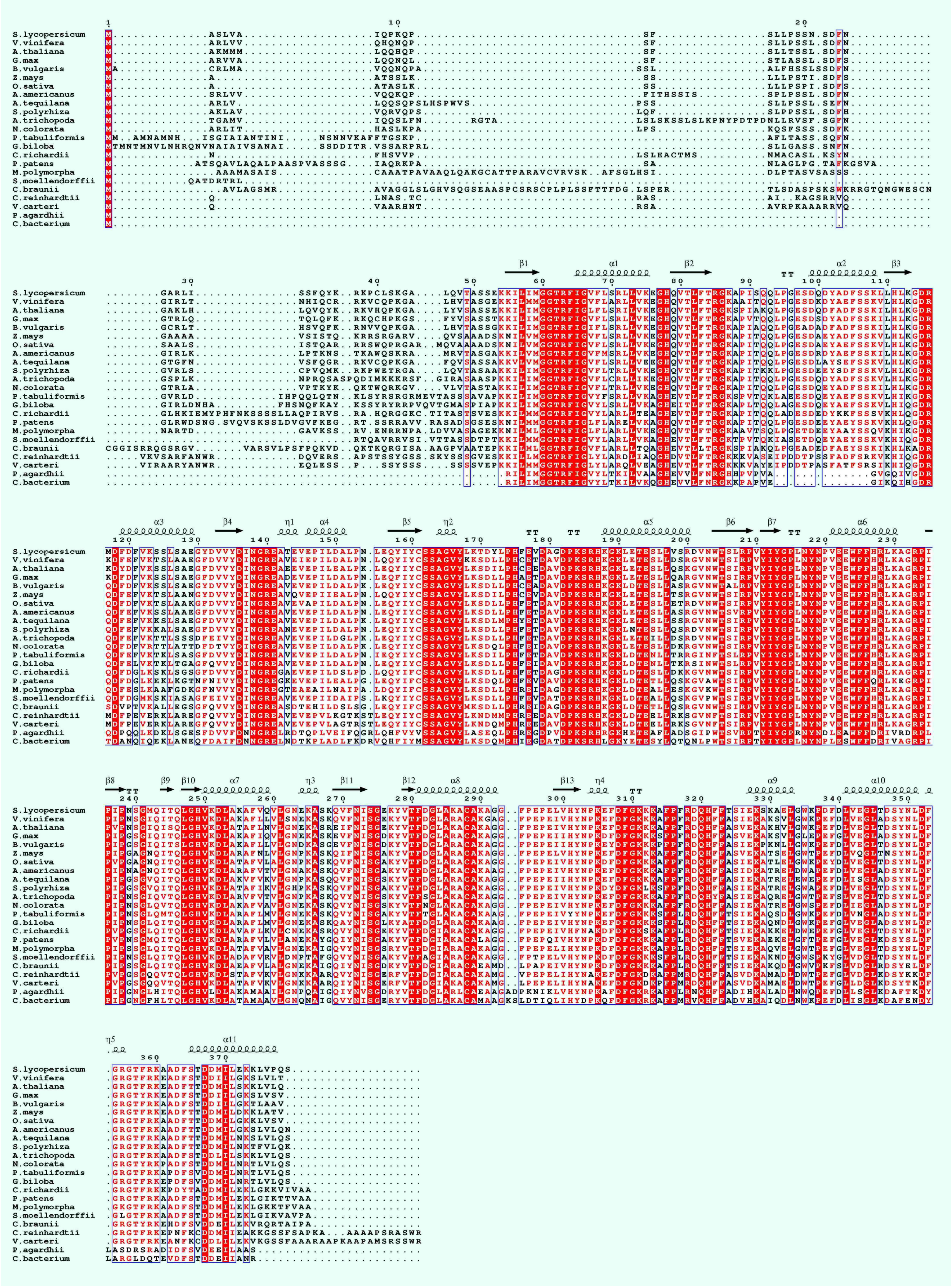

Figure S1: protein sequences used for constructing the phylogenetic tree of the CSP41b in photosynthetic organisms.

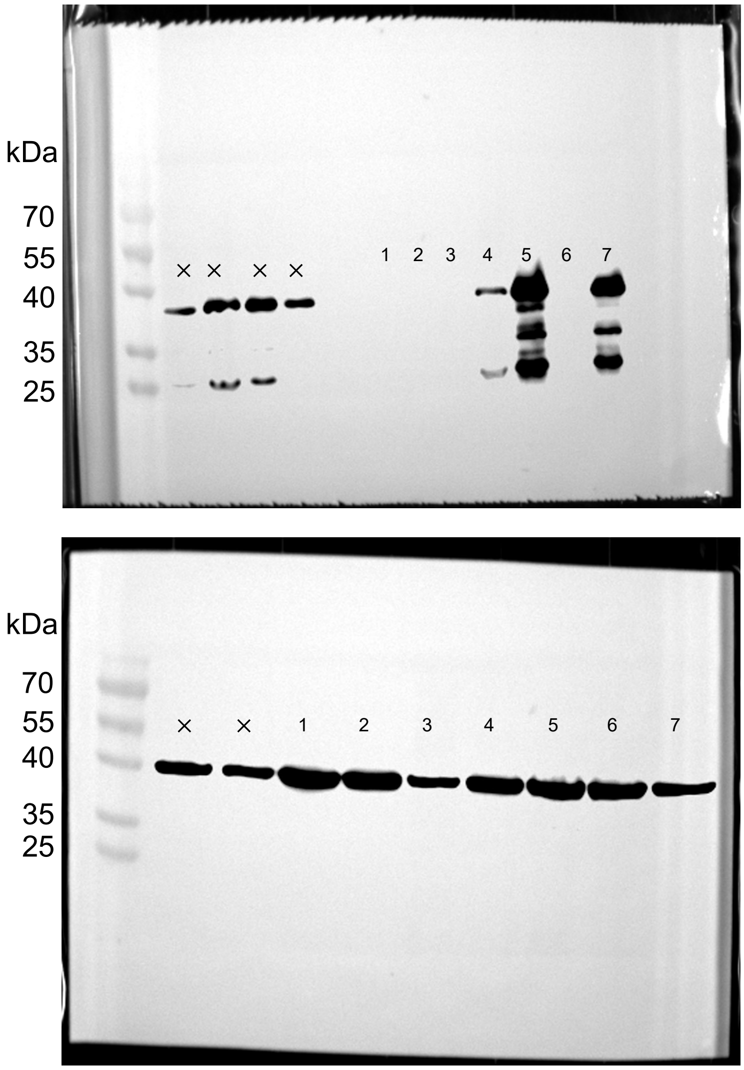

Figure S2. Expression CSP41b-FLAG protein in WT and *CSP41b-OE* plants. The upper panel shows Western blot results for CSP41b-FLAG, and the lower panel shows Actin1 controls. The molecular weight of the protein is 41.54 kDa. Lane 1: Wild Type; Lane 2, 3, 4, 6: Transgenic overexpression lines failed to detect target protein expression; Lane 5: CSP41b-OE line 1; Lane 7: CSP41b-OE line 2; Lane 6: Wild Type; Lane cross: Other materials.

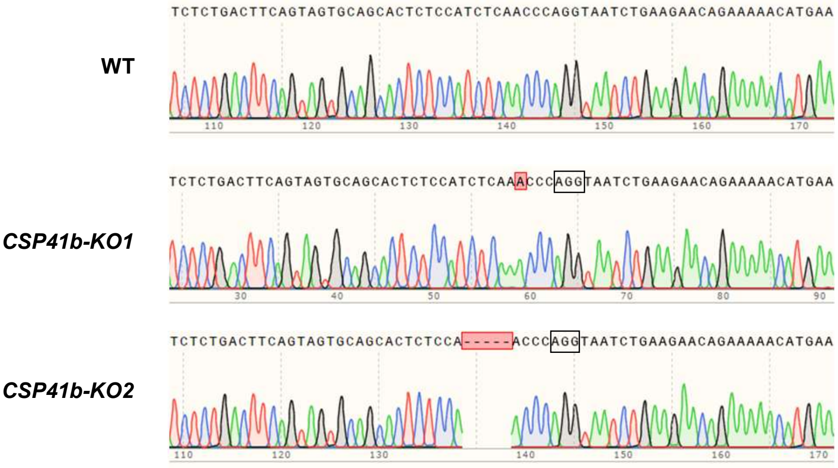

Figure S3: Sequencing chromatograms near the target sites of the two knockout lines of *CSP41b*.

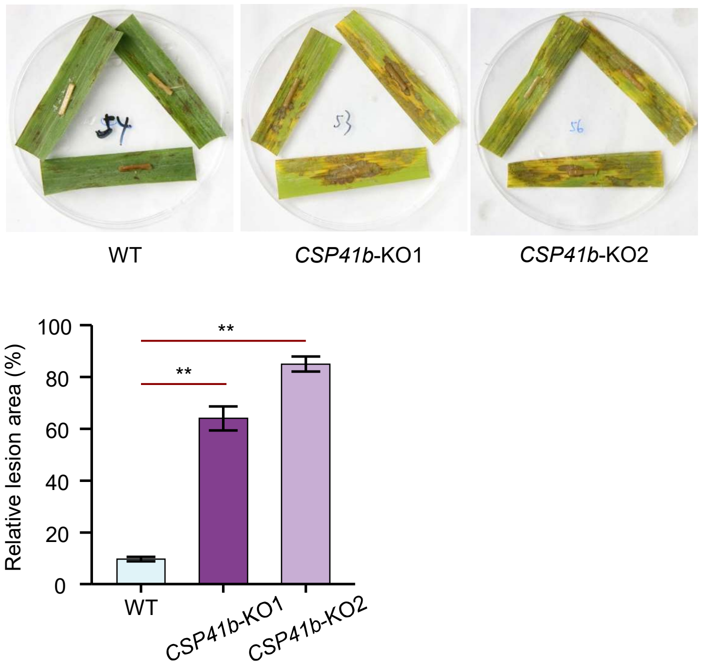

Figure S4: Relative lesion area measurement on detached leaves at 7 dpi (n = 3). Values are presented as mean ± SE. Significant differences were determined using a two-tailed Student's t-test (**$p < 0.01$).

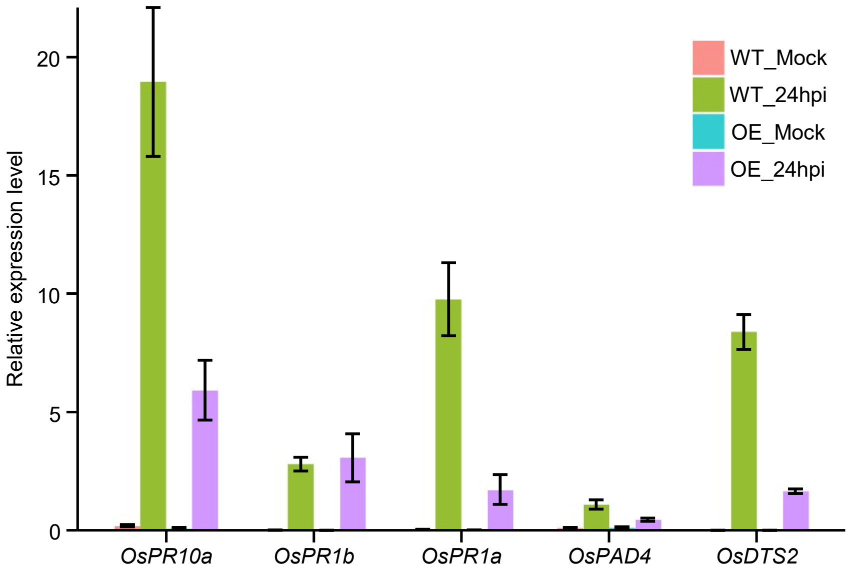

Figure S5: Five randomly selected pathogen-responsive rice genes were subjected to qPCR to validate the reliability of transcriptome data. These included: Three pathogenesis-related (PR) genes: *OsPR10a* (Os12g0555500), *OsPR1b* (Os01g0382000), and *OsPR10a* (Os07g0129200); Two phytoalexin biosynthesis-related genes: *OsPAD4* (Os11g0195500) and *OsDTS2* (Os04g0179700). The housekeeping gene *OsActin2* (Os11g0163100) served as the endogenous control. Biological samples were identical to those used for RNA-seq. Values are mean ±SE (n = 3).

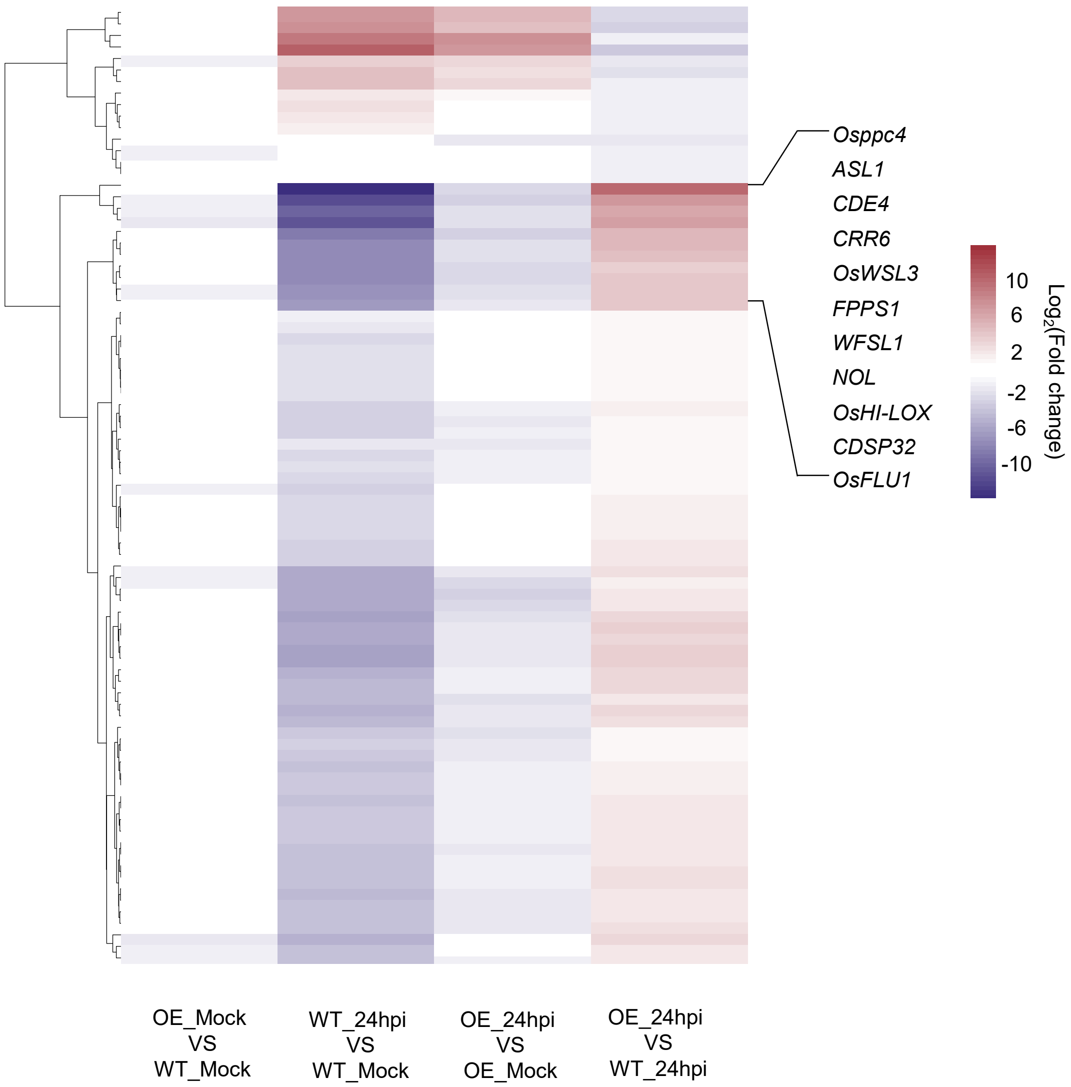

Figure S6: Heatmap of nuclear genome-encoded photosynthesis-related DEGs. Fold-change values are color-coded (red: upregulation; blue: downregulation).

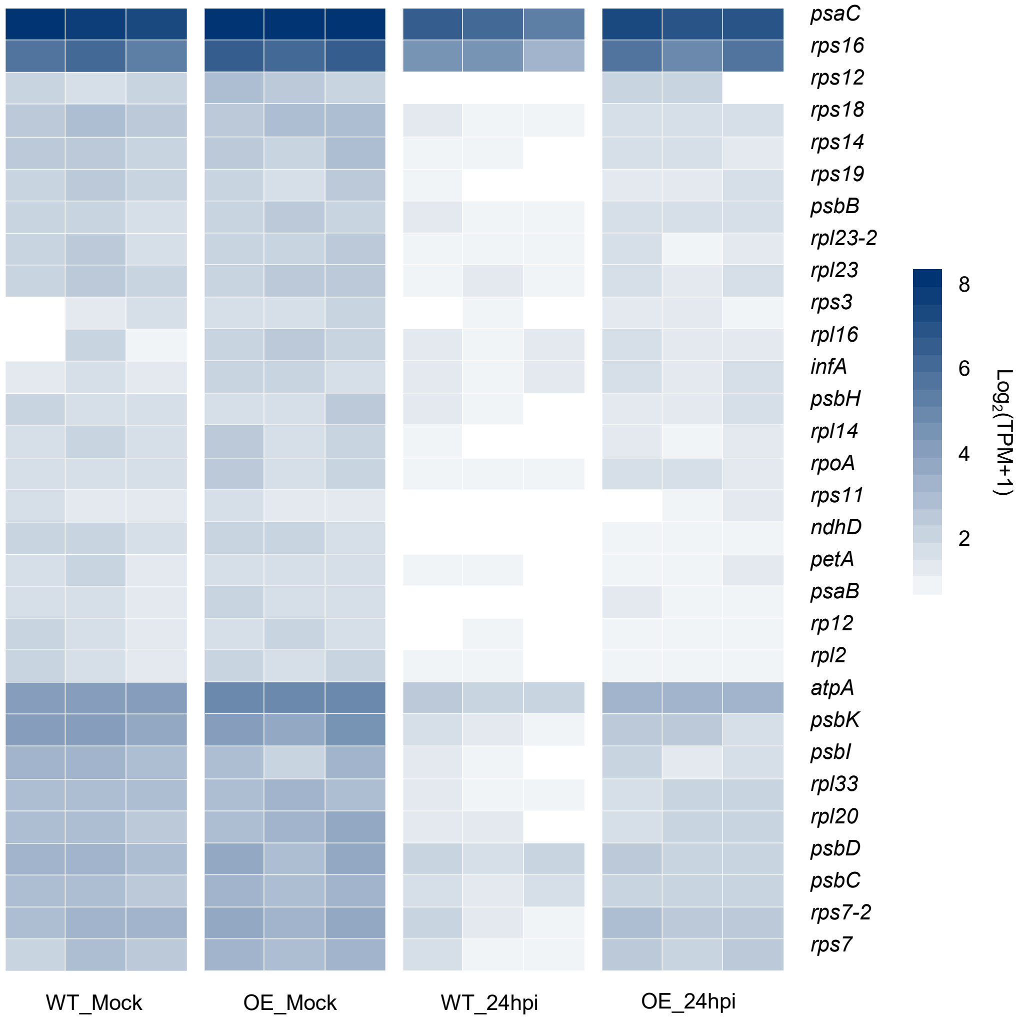

Figure S7: Heatmap showing DEGs mapped to the chloroplast genome across all samples. Expression levels are color-coded (blue: high expression; white: low expression).

Table S1: The primer sequence information involved in this article.

Primer name	Primer sequence(5′-3′)	Note
gCSP41b-F	GAAGAGCAGCCTCCTGCTAC	This primer pair is used to detect the knockout site of *OsCSP41b* by Sanger sequencing.
gCSP41b-R	CATGGCAGAAAAGCTAAGAG	
koCSP41b-F	AGCACTCTCCATCTCAACCCGTTTTAGAGCTAGAAATAGCAAGTTA	This primer pair is used for constructing the *OsCSP41b* knockout vector.
koCSP41b-R	GGGTTGAGATGGAGAGTGCTGCCACGGATCATCTGCACAAC	
cCSP41b-F	CATTTACGAACGATAGCCGGTACCATGGCAGCAACAGCCTCCCTGAAGAG	This primer serves as the forward primer for amplifying the coding sequence (CDS) of *OsCSP41b*, and is used for constructing both the overexpression vector and subcellular localization vector.
cCSP41b-gfp-R	CTGCAGGTCGACTCTAGAGGATCCGACGCTGACGAGCTTCTTGCCGAGGA	This primer serves as the reverse primer for amplifying the coding sequence (CDS) of *OsCSP41b* and is used for constructing the subcellular localization vector.
cCSP41b-flag-R	ATCATGATCTTTGTAATCGGATCCGACGCTGACGAGCTTCTTGCCGAGGA	This primer serves as the reverse primer for amplifying the coding sequence (CDS) of *OsCSP41b* and is used for constructing the overexpression vector.
qCSP41b-F	ACTTCAGTAGTGCAGCACTCTC	These primer pairs are used for RT-qPCR
qCSP41b-R	CTTGCCTGTCACCTTTCAAGTG	
q*OsPR10a*-F	TGTCCTAAAGTCGGATGTGCTC	
q*OsPR10a*-R	TTGAGCATGCCATAGTAGCCAT	
q*OsPR1a*-F	CTGTACTGTCAGCCGTATTTGC	
q*OsPR1a*-R	ACATGACCATCAGACCATGCAT	
q*OsPR1b*-F	GAGAAGAGCGACTACGACTACG	
q*OsPR1b*-R	ACGTACGCCCGTGTGTATAAAT	
q*OsDTS2*-F	AGAAATGGAATGTCGGTTCGGA	
q*OsDTS2*-R	GGGAATGTGAGGTTGAAGCCTA	
q*OsPAD4*-F	CGACTACCACCGAAACAACCTA	
q*OsPAD4*-R	GTTTCCACCACTTGTCGAACAG	
